# Animal modelling with the Francisella tularensis subspecies holarctica strain OR96-0246

**DOI:** 10.1099/mic.0.001637

**Published:** 2025-12-18

**Authors:** Kevin D. Mlynek, Sara I. Ruiz, Curtis R. Cline, Alexandra N. Jay, Ju Qiu‡, Ronald G. Toothman, Elsie E. Martinez, Wannaporn Ittiprasert†, Nancy A. Twenhafel, Joel A. Bozue

**Affiliations:** 1 Bacteriology Division, U.S. Army Medical Research Institute of Infectious Diseases (USAMRIID), Frederick, MD, USA; 2 Pathology Division, U.S. Army Medical Research Institute of Infectious Diseases (USAMRIID), Frederick, MD, USA; 3 Veterinary Medicine Division, U.S. Army Medical Research Institute of Infectious Diseases (USAMRIID), Frederick, MD, USA; 4 Regulated Research Administration Division, U.S. Army Medical Research Institute of Infectious Diseases (USAMRIID), Frederick, MD, USA

**Keywords:** animal modeling, *Francisella tularensis*, mouse, non-human primate, rat, tularemia, type B

## Abstract

Tularemia is a zoonotic disease caused by *Francisella tularensis*. Most human cases are caused by *F. tularensis* ssp. *tularensis* (type A) or *F. tularensis* ssp. *holarctica* (type B), with the former considered more virulent. For this reason, type A isolates are often the benchmark for the testing of new vaccines or antibiotics. However, both subspecies cause considerable disease and can differ in their responsiveness to medical countermeasures. Accordingly, there is a need to identify and characterize representative type B isolates that are available to qualified research institutions to ensure the development of future vaccines or antibiotics is efficacious against both subspecies. The type B isolate OR96-0246 was identified as a strain that can address this need and was subsequently characterized. For *in vitro* characterization, the OR96-0246 strain was examined for growth in media and for its ability to form biofilm. As the LPS is an essential virulence factor, the O-antigen was characterized through western analysis. For future medical countermeasure testing for biodefence concerns, pneumonic challenges with animal modelling would be required. Therefore, using the OR96-0246 strain, we implemented animal models that encompassed BALB/c mice, Fischer 344 rats and cynomolgus macaques. Mice were challenged via intranasal instillation with varying doses of OR96-0246, and the LD_50_ was determined to be 1 c.f.u. We progressed to Fischer 344 rats, which are a better-suited rodent model to gauge vaccine efficacy. When challenging the rats by whole body aerosolization with various doses of OR96-0246, the LD_50_ was determined to be 138 c.f.u. Finally, a staircase challenge design was applied to three cynomolgus macaques, each receiving a different aerosolized dose of OR96-0246 to determine an estimated LD_50_ for non-human primates (NHPs). Two out of the three NHPs succumbed to the challenge. The animal that received the lowest dose (2.1×10^4^ c.f.u.) survived but did demonstrate clinical signs of infection. Samples from the challenged rats and NHPs were collected for histopathology characterization. Generally, the pathological changes observed in both models were similar, consisting primarily of multifocal bronchopneumonia in the lung and necrotic lesions in the spleen. This animal model development with type B strains of *F. tularensis* will be essential to properly evaluate new antimicrobials and vaccines to protect against tularemia.

## Data Availability

All data generated or analyzed during this study are included in this published article and its supplementary information files.

## Introduction

Tularemia is a life-threatening zoonotic disease caused by the Gram-negative intracellular bacterium *Francisella tularensis*. This species is divided into multiple subspecies, with each subspecies differing in geography, vector ecology and virulence. *F. tularensis* ssp. *tularensis* (type A) and *F. tularensis* ssp. *holarctica* (type B) are responsible for most human tularemia cases and are considered Tier 1 select agents in the USA due to a low infectious dose, high morbidity and the risk of aerosol exposure [1]. Subspecies are associated with different geographies, as type A isolates are found only in North America, while type B isolates are typically distributed throughout the northern hemisphere [2,9]. However, in Australia, tularemia cases have been reported following interactions with common ringtail possums, indicating the emergence or increased detection of type B strains beyond just the northern hemisphere [10,11].

Further, infection ecology differs as type A isolates tend to be associated with tick vectors and lagomorph populations [3]. In contrast, type B isolates are most often associated with a mosquito vector and rodents in close proximity to a water source [3,12, 13]. Ticks have also been shown to carry type B strains and lead to disease in humans [12,14]. As *F. tularensis* is a zoonotic disease, it has been shown to infect >100 species, which are typically distributed throughout the northern hemisphere [15,17]. However, as stated above, recent cases have detected tularemia outside of the normal expected regions, suggesting a more globalized expansion of *F. tularensis* [10,11, 18, 19].

Historically, type A isolates are considered more virulent than type B, though both can cause considerable disease or death [20]. Infections caused by type B isolates have increased in recent years and could represent an emerging threat, particularly in light of evolving vector dynamics [21,23]. Disease onset typically occurs as an acute febrile illness with a variety of clinical presentations, often associated with the route of pathogen entry [1,13, 24]. The most common form of tularemia is glandular and ulceroglandular, introduced from an insect bite or direct contact [24,26]. Oropharyngeal tularemia has received increasing attention as disease epidemiology shifts from sporadic individual cases to small, localized outbreaks due to a shared contaminated water source [27,28]. One of the most severe forms of disease, pneumonic tularemia, can occur from clinical progression of other forms or direct inhalation of *F. tularensis* and is the greatest concern from a public health standpoint [1,29]. Typhoidal tularemia due to sepsis or bacteraemia would also result in a severe form of the disease. With this in mind, there are continued efforts placed on novel vaccine and antibiotic development to combat human *F. tularensis* infection, with emphasis placed on the more lethal pneumonic forms of disease.

Animal model development is essential to properly evaluate bacterial pathogenesis as well as the efficacy of medical countermeasures, such as antimicrobials and vaccines. Tularemia has been modelled in multiple mammalian animal species (reviewed in [30,31]), including mice [32,34], rats [35,36], hamsters [37], guinea pigs [33], rabbits [33,38], non-human primates [39,41] and even humans [42,44]. It is generally agreed upon that a single animal model is not sufficient to gather data necessary to advance all forms of medical countermeasures against tularemia [30]. While mice are a cost-effective and well-established animal model, they are highly susceptible to *F. tularensis* infection, as 1 c.f.u. is typically lethal, including subspecies that do not normally cause disease in humans [45]. For this reason, mice are an excellent model to detect virulence defects but are often a challenging model to accurately assess medical countermeasures. Rats have been proposed as a more appropriate rodent model to bridge this gap, as they exhibit similar sensitivity as humans to both *F. tularensis* type A and B isolates, which can be particularly useful for screening new vaccines or therapeutics [36,46,50]. Early studies demonstrated that guinea pigs and rabbits are problematic for tularemia models for product development, as sensitivity to infection differs greatly between type A and B isolates in these animals [33]. Non-human primates (NHPs) are arguably the most comparable to humans from an anatomical standpoint and mirror many of the hallmark features noted in clinical cases [39,40]. NHP models have been successful in both vaccine and therapeutic testing; however, these studies carry significant costs and ethical considerations.

The prototypical virulent *F. tularensis* isolate for this species, Schu S4, is of type A lineage and is perhaps the most studied fully virulent strain to date, with most modelling and medical countermeasures tested to demonstrate efficacy. Still, there is a need to identify and characterize representative type B isolates that are available to qualified research institutions to ensure the development of future vaccines or antibiotics that are efficacious against both type A and B isolates, particularly in light of known virulence differences between type A and B isolates. Successful tularemia medical countermeasures need to protect against a wide variety of *F. tularensis* strains, which may differ in geographic origin or virulence attributes. We previously developed a well-characterized panel of *F. tularensis* strains, which represents a variety of historical, clinical and environmental isolates but needs better representation of type B strains [51]. For this reason, we assessed the *in vitro* characteristics and virulence in various animal models of the type B isolate OR96-0246. This strain was previously isolated from a monkey in Oregon, USA, in 1996 and clusters within the B4 subclade [52,55].

## Methods

### Bacterial strains and growth media

The OR96-0246 strain of *F. tularensis* ssp. *holarctica* was obtained from Biodefense and Emerging Infections Resources (BEI) (https://www.beiresources.org/), catalogue #NR-648. Comparator *F. tularensis* strains used in this study were Schu S4, a prototype type A strain (obtained from BEI Resources; catalogue #NR-10492) [56], and FRAN255, a type B strain originally collected from a human clinical case in Kentucky [51]. *F. tularensis* strains were grown at 37 °C on chocolate agar plates (Remel). For broth-grown cultures, strains were grown in brain heart infusion (BHI) broth (Becton Dickinson) supplemented with IsovitaleX (Becton Dickinson) as indicated or in Chamberlain’s defined medium (CDM) [57] (Teknova).

### Growth analysis

Bacterial strains were suspended in PBS (pH 7.2) to an OD_600_ of 0.3. Bacterial suspensions were diluted 1 to 10 into either BHI supplemented with IsoVitaleX as indicated or CDM in a CoStar polystyrene 96-well plate. Growth was then assayed by OD_600_ reading every 30 min using a Tecan Spark (Tecan Systems) microplate reader at 37 °C with orbital shaking. Absorbance values were determined using the average of triplicate wells and subtracting the medium background as determined by the sterility control. All data reported are the result of at least three individual experiments.

### Biofilm analysis

Determination of biofilm production from *F. tularensis* strains was performed as previously described [58]. Bacterial suspensions from a plate-grown culture were diluted in CDM, seeded into 96-well plates, and statically incubated at 37 °C for the indicated length of time. Sterility wells were included in each experiment, and peripheral wells were avoided to minimize edge effects. Prior to biofilm staining, the OD_600_ was measured, after which plates were aspirated, washed 3× with PBS to remove planktonic cells and fixed with 100% ethanol for 30 min at room temperature. Following ethanol fixation, 0.1% crystal violet (w/v) was added to each well for 15 min and washed 3× with PBS, after which the remaining crystal violet stain was solubilized in 33% acetic acid. The OD_600_ was measured to quantify crystal violet staining as an indicator of biofilm formation. When necessary, samples were diluted 1 :10 in 33% acetic acid to ensure that OD readings were within the linear range. At least four technical replicates were averaged in each experiment. All data reported are the result of at least three individual experiments. Where referred to in text, wells were considered biofilm positive if crystal violet staining was at least twofold higher than the sterility control wells, with this background subtracted. For instance, if the sterility control wells had an OD_600_ value of 0.25 after staining, an experimental well would be required to have a value of at least 0.5 after subtracting the sterility control to be considered biofilm positive.

### Western blot analysis of the O-antigen

Representative *F. tularensis* strains (OR96-0246, Schu S4 and FRAN255) were resuspended in PBS to an OD_600_ of 0.5 (equivalent to ~10^9^ c.f.u. ml^−1^), and 1 ml aliquots were used to prepare whole cell extracts. Cell pellets were suspended in 1× NuPage gel loading buffer (Thermo Fisher), boiled for at least 45 min and confirmed to be inactivated. Samples (~10^7^ c.f.u.) were size separated on a NuPage Novex 4–12% Bis-Tris gel and transferred to a nitrocellulose membrane using an iBlot Gel Transfer Device (Thermo Fisher). Membranes were blocked with 5% skim milk in Tris-buffered saline +0.5% Tween 20. Membranes were probed with mouse anti-LPS monoclonal antibody (FB-11) (Thermo Fisher), anti-capsule antibody (11B7) [59] or rabbit anti-GroEL polyclonal antibody (Enzo Life Sciences). The antibodies were detected using horseradish peroxidase-conjugated goat polyclonal anti-mouse (FB-11, 11B7) or anti-rabbit (GroEL) secondary antibodies at 1:5,000 dilution, respectively. Bands were visualized using a Clarity Max ECL Western Blotting Substrate Kit (Bio-Rad Laboratories) following the manufacturer’s directions.

### Animal modelling

#### Murine intranasal LD_50_ determination

Female BALB/c mice (6–8 weeks old) were obtained from Charles River Laboratories and allowed a week to acclimate. The LD_50_ for the OR96-0246 strain was determined by intranasal challenge as previously described [51,60]. Briefly, the titre of the challenge dose was determined by serial dilutions of OR96-0246 in PBS and plated on chocolate agar for c.f.u. counts. Mice were anesthetized with 150 µl of ketamine, acepromazine and xylazine injected intraperitoneally. OR96-0246 was suspended in PBS to an OD_600_ of ~0.5 from swabbed plate cultures grown for 24 h and tenfold serially diluted. Mice (*n*=10/group) were then challenged by intranasal instillation with 50 µl of the diluted inoculum at the indicated concentrations with five different doses of *F. tularensis*. Challenged mice were observed at least daily for 14 days for clinical signs of illness. Observations increased when clinical signs occurred. Humane endpoints were used, and mice were euthanized when moribund according to an endpoint score sheet.

#### Fisher rat aerosol exposure LD_50_ determination

Female Fischer 344 rats (6–8 weeks old) were obtained from Charles River Laboratories and allowed a week to acclimate prior to use. The method used to determine aerosol LD_50_ in the rat model was previously described [36]. Briefly, OR96-0246 was prepared from CDM cultures grown overnight in a 37 °C shaker at 200 r.p.m. The cultures were adjusted for five different challenge doses, and the c.f.u. of the starting cultures were determined by plating on chocolate agar. Aerosolized doses of OR96-0246 were administered to each challenge group (*n*=8) using a dynamic 30 litre humidity-controlled Plexiglas whole-body exposure chamber, as previously described [61]. An estimate of the inhaled dose was obtained as previously described [62,64]. Briefly, the generated aerosol was sampled for the duration of the exposure with a 6 l min^−1^ all-glass impinger (AGI) (part #7541–10, Ace Glass Inc., Vineland, NJ). AGIs contained 10 ml of BHI broth and 40 µl of an antifoaming agent. The samples were then serially diluted and plated onto chocolate agar plates for c.f.u. determination. The inhaled doses were determined by calculations as previously described [65]. Rats were monitored daily, and mortality rates (to include euthanasia when moribund in accordance with early endpoint criteria) were recorded for 21 days.

#### NHP modelling by aerosol exposure

Three Cynomolgus macaques (*Macaca fascicularis*) (CMs) weighing ≥3 kg were used in this study. All NHPs underwent a physical exam and pre-operative bloodwork for a complete blood count and blood chemistry panel that included, at a minimum, kidney and liver function within 30 days of telemetry implantation to measure body temperature and activity. Each NHP was exposed (head only) with aerosolized OR96-0246 using a Collison Nebulizer to produce a highly respirable aerosol (flow rate 7.5±1 l/min). A staircase model design was utilized to conserve NHP numbers. Bacterial challenge material was grown as previously described in BHI supplemented with IsovitaleX [36]. Aerosol doses were calculated as described above for the Fischer rats.

Following the challenge, NHPs were monitored at least once per day for clinical signs of disease for 20 days post-exposure (DPE). Observations increased when clinical signs occurred. Cage side observations included recording respiratory rate by counting respirations for 15 s for each animal at least once per day. Baseline was determined pre-challenge from respiratory rates at days 3, 2 and 1, then averaged to generate individual baselines for each animal.

#### Telemetry in the NHP model

Telemetry implants (Data Sciences International, Inc. (DSI)) were used to detect and measure temperature and activity in subject animals (model M00). Subjects were placed in standard cages with transceivers (TRX, DSI) mounted in close proximity. Temperature was collected using a sampling rate of one sample per second, and data were analysed for all subjects individually. Data reduction was performed in 30-min intervals for temperature.

#### Radiographs in the NHP model

Ventrodorsal and lateral thoracic radiographs were acquired on anesthetized NHPs (Telazol, Zoetis, Kalamazoo, Michigan; 3 mg/kg) using digital radiography (MinXray TR90, Northbrook, Illinois; 72 kVp, 1.80 mAs). Images were scored in comparison to baseline from 0 to 18 by evaluating cranial, middle and caudal lung segments.

#### Histopathology assessment

For NHPs, post-mortem tissues were collected, and samples were weighed, homogenized and plated on chocolate agar to determine if *F. tularensis* was present. For histopathology analysis for rats and NHPs, tissues were fixed in 10% neutral buffered formalin, embedded in paraffin and sectioned for haematoxylin and eosin (H and E) staining. At least a single section of the above tissues was examined by a board certified veterinary pathologist (unblinded) and was subjectively graded on the severity of necrosis/inflammation: minimal (involving *<*10% of the tissue), mild (involving 11–25% of the tissue), moderate (involving 26±50% of the tissue), marked (involving 51±79% of the tissue) or severe (involving *>*80% of the tissue). Additionally, immunohistochemistry (IHC) analysis using a BOND RX Automated Stainer (Leica Biosystems) was performed to identify *F. tularensis* localization in samples. Tissues were scored for *F. tularensis* IHC positivity using a mAb mouse anti-*F. tularensis* LPS (F6070-02X; US Biological) at 1:5,000.

#### Statistics

For growth assays, a logistic curve was fit [66], which yielded estimates of the lag time, max growth rate and asymptote. The significance of pairwise group comparisons was obtained from a linear mixed-effects model. In biofilm assays, group differences in positivity frequency were compared using the chi-square test. The LD_50_ was estimated by Probit analysis or Reed–Muench method, using log10 dose as the predictor. Median time to death (TTD) and accompanying confidence limits were estimated by Kaplan–Meier survival methods. Respiratory rate was analysed via two-way ANOVA in PRISM version 10.1.2 (GraphPad). For NHP blood analysis, data were analysed by a linear mixed effects model. The multiplicity was adjusted by Dunnett’s method. Analysis is implemented in SAS version 9.4 (SAS Institute Inc.).

## Results

### *In vitro* growth analysis of OR96-0246

**Fig. 1.**
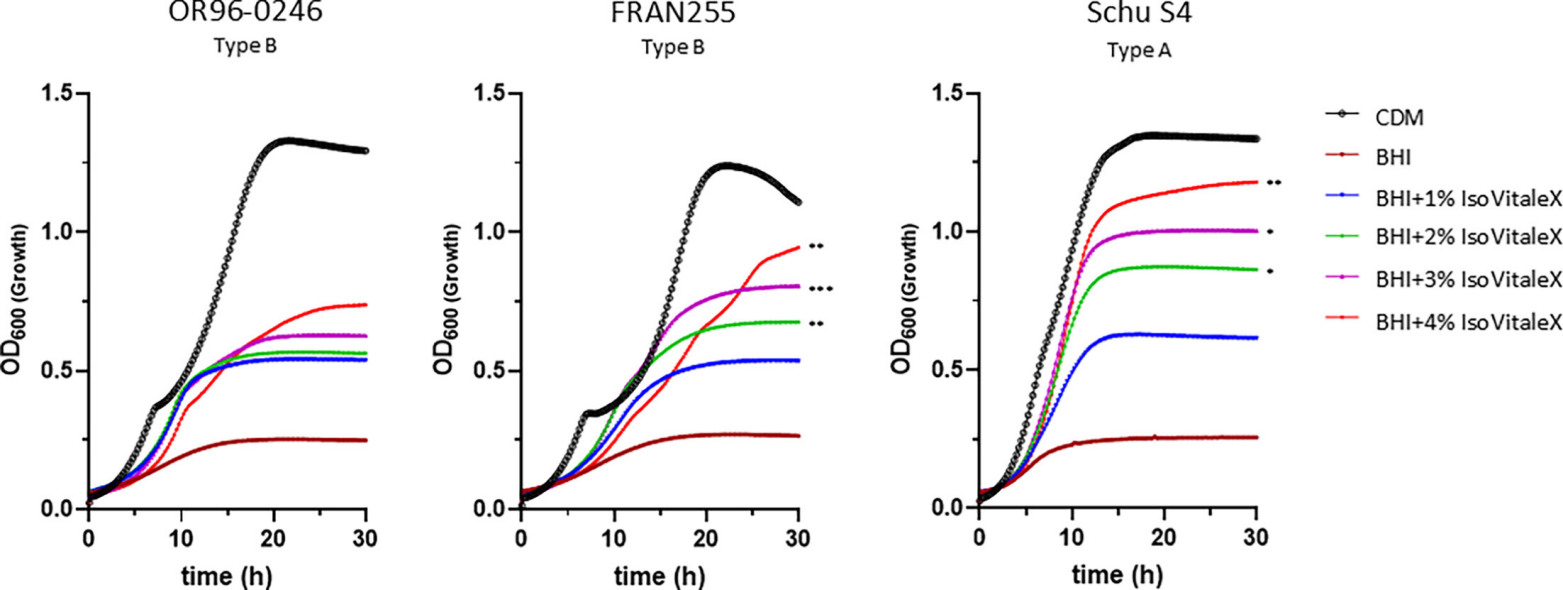
*In vitro* growth characteristics of *F. tularensis*. Three different strains of *F. tularensis* (OR96-0246 (type B), FRAN255 (type B) or Schu S4 (type A) were grown in CDM or BHI broth supplemented with a varying amount of IsoVitaleX as indicated and grown at 37 °C. Optical density (OD_600_) measurements were based upon quadruplicate technical replicates, and the experiment was repeated three times independently. Standard deviation bars are omitted for clarity. *P<0.05; **P<0.01; ***P<0.001 compared to BHI+1% IsoVitaleX using pairwise group comparisons obtained from a linear mixed-effects model.

*F. tularensis* is routinely grown in CDM, a nutritionally defined medium, or BHI supplemented with 1% IsoVitaleX, a complex medium that promotes a phenotypically host-adapted state [67]. While Schu S4 can grow robustly in both CDM and BHI, it has been observed that fully virulent strains sometimes fail to reach a similar density when grown in supplemented BHI compared to Schu S4 [51]. To define the planktonic *in vitro* growth patterns of OR96-0246, we performed growth curve analysis using CDM and BHI supplemented with increasing concentrations of IsoVitaleX using Schu S4 (type A) and FRAN255 (type B) as references. Similar to Schu S4 and FRAN255, OR96-0246 reached an OD_600_ density >1.0 within 24 h in CDM, with a slight inflection at ~ 6–8 h post-inoculation, similar to FRAN255, which has been observed with other type B isolates (Fig. 1).

All *F. tularensis* strains required additional supplementation and failed to grow appreciably in BHI without enrichment. When grown in BHI+1% IsoVitaleX, OR96-0246 exhibited a similar growth pattern as observed with the reference strains. However, both FRAN255 and Schu S4 had significant increases in the growth rate with supplementation beyond 1%, while OR96-0246 lacked a significant response but showed this trend (Fig. 1).

### O-antigen profiling and biofilm formation of OR96-0246

**Fig. 2.**
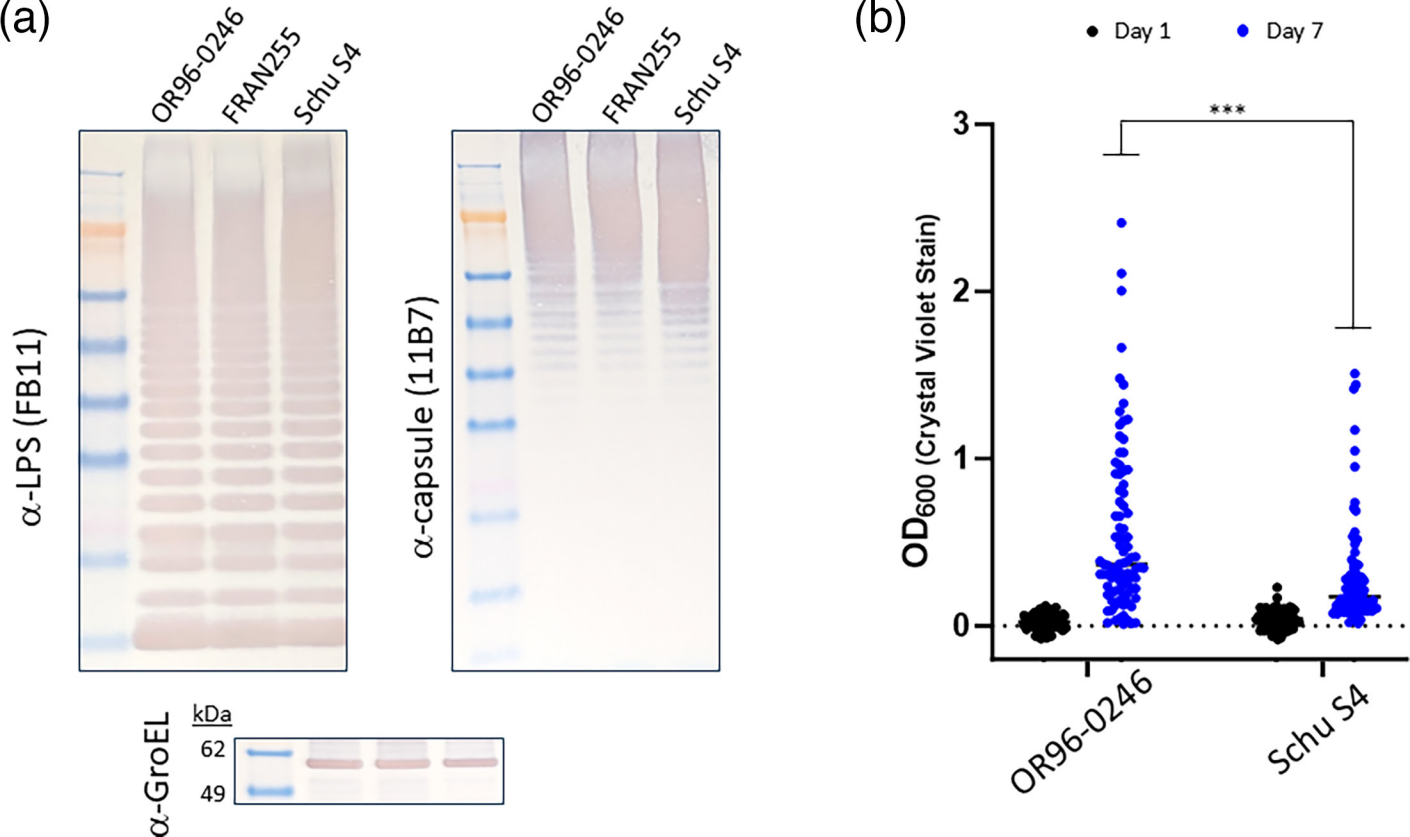
Analysis of the O-antigen and biofilm formation of *F. tularensis* strain OR96-0246. (**a**) Whole cell extracts were run on SDS-PAGE gels at equal concentrations and blotted with either a monoclonal antibody to the O-antigen of LPS (left) or capsule (right) of the listed *F. tularensis* strains. Equal loading of sample material was demonstrated when blotting the extracts with a polyclonal antibody directed against the GroEL protein. Molecular masses are indicated on the left in kDa. (**b**) Biofilm formation for OR96-0246 and Schu S4 was assessed by crystal violet staining at days 1 and 7 post-inoculation. Each point graphed represents an individual well in an experiment. The black bar indicates the median value after at least three independent experiments. ***P<0.001 assessing group differences in positivity frequency by chi-square test.

The *F. tularensis* O-antigen presented on the cell surface, predominantly in the form of LPS and capsule, is an important virulence determinant in *F. tularensis* as it helps subvert host immune detection and is required for virulence [68,71]. To determine if OR96-0246 possesses the canonical O-antigen profile, we performed Western blotting using whole cell extracts of this strain and compared it to FRAN255 (type B) and the prototype Schu S4 (type A), both of which we have previously examined [51]. This experiment demonstrated that the O-antigen produced by OR96-0246 is similar to the profile of other *F. tularensis* strains for both LPS and capsule (Fig. 2a).

Antigenic variation of the LPS and O-antigen has been observed, though the exact mechanism or function of this phenomenon is unknown [72,73]. We previously showed that biofilm formation occurs in a stochastic manner dependent upon spontaneous mutations of LPS biosynthesis genes, with type B isolates more readily varying in variant formation [74,75]. To determine the propensity of phase variation and the subsequent biofilm formation of OR96-0246, biofilm was measured at 1 and 7 days for OR96-0246 (type B) and Schu S4 (type A). These results demonstrated that indeed OR96-0246 is able to undergo variation to a biofilm state at a higher frequency than Schu S4 (*P*<0.001), which is consistent with other type B isolates we have previously characterized [58] (Fig. 2b).

### Small animal modelling of OR96-0246 pathogenesis

**Fig. 3.**
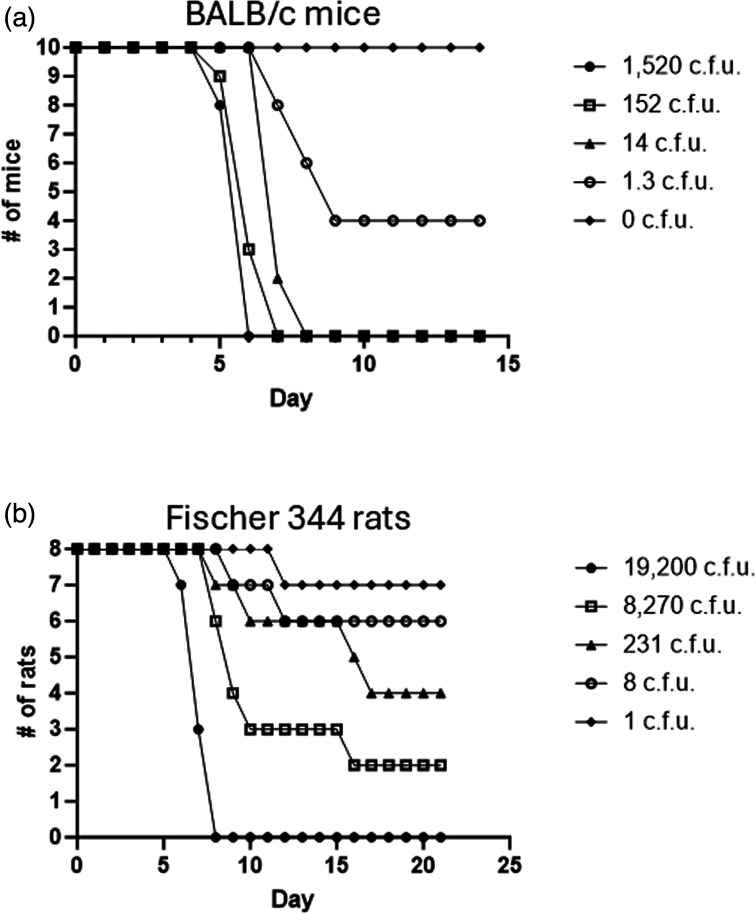
Small animal modelling of pneumonic tularemia and LD_50_ analysis following challenge with OR96-0246. (**a**) Survival data of BALB/c mice (groups of *n*=10) were challenged intranasally, or (b) Fischer 344 rats (groups of *n*=8) were challenged by whole body aerosolization with the OR96-0246 strain of *F. tularensis*. Survival was monitored following infection to determine the LD_50_ for each rodent model. LD_50_ estimates reported in the text were calculated from these data by Probit analysis.

Though mice are exquisitely sensitive to *F. tularensis*, a murine model is important not only to initially assess the virulence of *F. tularensis* isolates but also to test genetically derived mutant strains for attenuation. Furthermore, mice can serve as a cost-effective *in vivo* model for initial assessment of medical countermeasure before progressing to higher animal models. To establish pneumonic LD_50_ values of OR96-0246, BALB/c mice were challenged intranasally with doses ranging from ~0 to 1500 c.f.u., and survival was followed for 14 days (Fig. 3a). The LD_50_ at 14 days post-challenge was determined to be <1 c.f.u. The median TTD was determined to be 6 days at the higher challenge doses (152 c.f.u. and 1,520 c.f.u.) and extended to 9 days at ~1 c.f.u. (Table 1). The results determined here for OR96-0246 are in agreement with the mouse virulence studies we performed previously with a diverse panel of *F. tularensis* strains [51]. These data demonstrate that OR96-0246 is a virulent type B isolate that offers a window of opportunity for utilization in post-challenge intervention studies in a tularemia mouse model.

**Table 1.** Time to death analysis of rodent models challenged with OR96-0246

Species	Dose (c.f.u.)	TTD median (95% CL)*	TTD mean (se)^†^
	0	>14	>14
	1.3	9.0 (7.0, >14)	8.4 (0.3)
**BALB/c mice** (intranasal)	14	7.0 (>14, >14)	7.2 (0.1)
	152	6.0 (5.0, 7.0)	6.2 (0.2)
	1,520	6.0 (5.0, 6.0)	5.8 (0.1)
**Fischer 344 rats** (aerosol)	1	>21 (12.0, >21)	12
	8	>21 (9.0, >21)	11.6 (0.5)
	231	>21 (8.0, >21)	14.9 (1.4)
	8,270	9.5 (8.0, >21)	11.5 (1.4)
	19,200	7.0 (6.0, 8.0)	7.3 (0.3)

* CL confidence level; †se standard error.

 Rats have been identified as an appropriate small animal model to perform vaccine testing for *F. tularensis*, as they closely reflect human susceptibility and are resistant to challenge with *Francisella* species not considered pathogenic for higher animals [32,35, 46]. To determine the LD_50_ value of the OR96-0246 strain, we performed whole-body aerosol exposures at five different challenge doses (1, 8, 231, 8,270, and 19,200 c.f.u.). The challenged rats were followed for 21 DPE. From this survival study, it was determined that the LD_50_ value for this strain of *F. tularensis* was 138 c.f.u. (Fig. 3b). The TTD measurements for the various challenge doses were calculated and included in Table 1.

### Pathology of Fischer 344 rats challenged by aerosol with OR96-0246

**Fig. 4.**
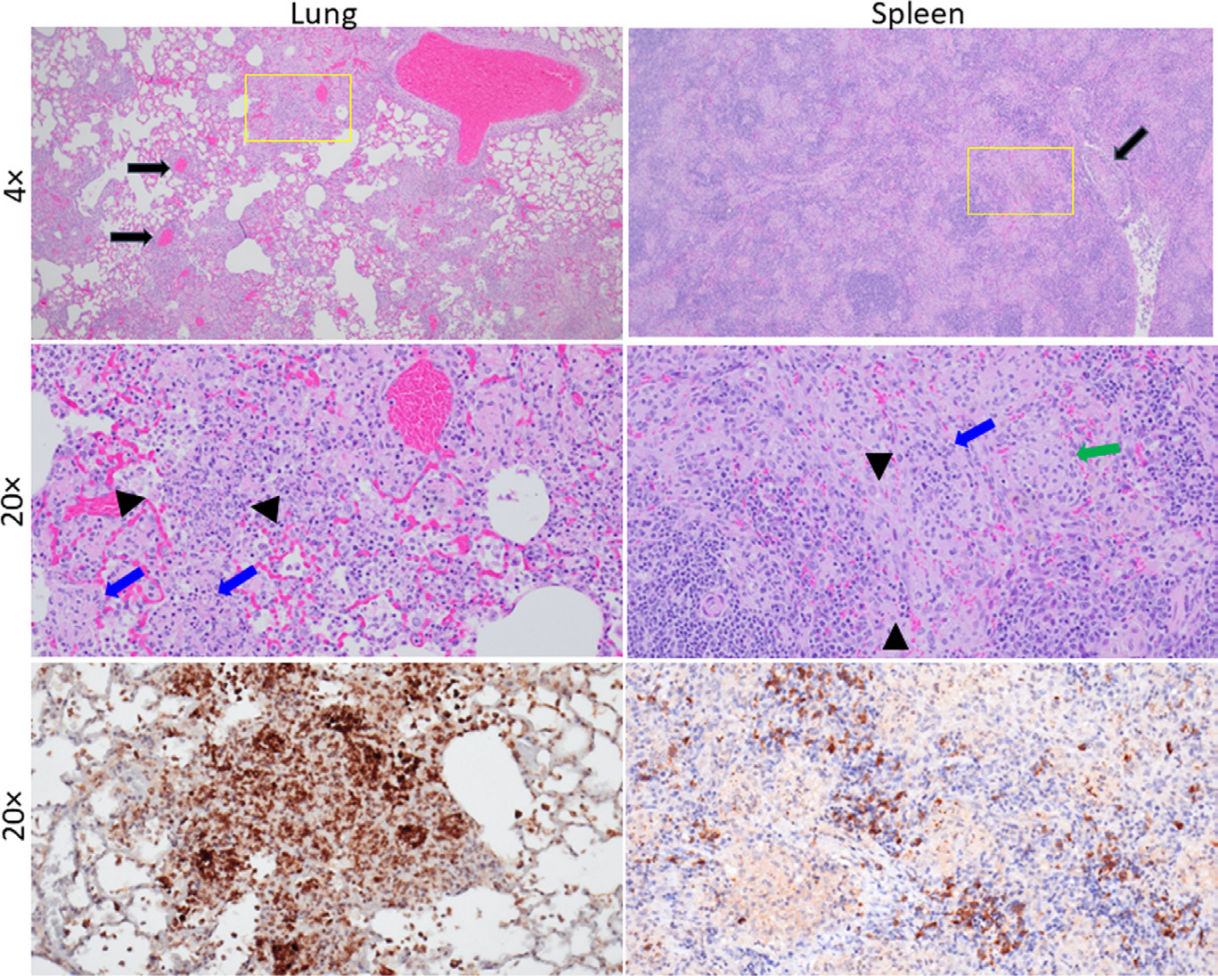
Histopathological analysis of OR96-0246 aerosol-challenged Fischer 344 rats. Representative images are from a rat that succumbed to a challenge with 8,270 c.f.u. Samples shown are from the lung and spleen. The upper images are at 4× magnification. The images immediately underneath the 4× images are 20× magnification from areas as indicated in the yellow box. Lungs: 4× image top. There are multifocal to coalescing areas of inflammation and necrosis throughout the section, as well as perivascular inflammation in the majority of vessels, both large and small calibre (black arrows). 20× image middle. There is alveolar necrosis (between black arrowheads) and inflammation in thickened and consolidated alveoli and accumulation of dense polymerized fibrin (blue arrows), oedema and necrotic debris. 20× image bottom. Representative image from the rat lung with IHC positivity indicating the presence of *F. tularensis*. Spleen. 4× image. There is severe lymphoid depletion in the white pulp and multifocal areas of pallor throughout the red pulp, which represent foci of inflammation and lytic necrosis. There is also an accumulation of fibrin and fibrin thrombi (black arrow). 20× image. There are multifocal areas of lytic necrosis and inflammation in the red pulp (blue arrow). There is also lymphoid depletion in the white pulp (marginal zone prominently) at the bottom left (between black arrowheads) and deposition of fibrin (green arrow). 20× image bottom. Representative image from the rat spleen with IHC positivity indicating the presence of *F. tularensis*.

The lungs and spleens of multiple rats that succumbed to disease or survived till the end of the study were analysed for histopathological changes. Overall, the pathology observed for rats challenged with OR96-0246 is similar to previous observations of rats exposed to Schu S4 [36]. For this particular study with the type B OR96-0246 strain, the most significant lesions in all challenge groups for the lung tissue include alveolar necrosis and inflammation that appeared in a multifocal to coalescing patchy pattern in the majority of lobes (Fig. 4, left). Foci of inflammation and necrosis were located throughout the lung from the central areas adjacent to or surrounding bronchi to the lobe periphery. Severity of alveolar necrosis ranged from minimal to marked, while severity of alveolar inflammation ranged from mild to severe and showed a dose-dependent effect, with inflammation most severe in the highest dose group (c.f.u. 19,200). Necrosis was most severe in the second to highest dose group (c.f.u. 8,270) (Table 2). The inflammation was mononuclear and neutrophilic, and there were variable amounts of fibrin and oedema multifocally filling alveolar lumina (exudate). Alveoli filled with a dense fibrin exudate often appeared confluent with adjacent lumina and septa, imparting a consolidated appearance in the most severe areas. There was also minimal to moderate, predominantly mononuclear, perivascular inflammation surrounding small to intermediate-sized vessels. Other lung lesions that were seen in a subset of animals that succumbed to disease include minimal or mild degeneration and necrosis of bronchiole epithelium in the two high-dose groups, minimal to mild pleuritis, minimal to mild peri-bronchial inflammation, bronchiole exudate, BALT hyperplasia and type II pneumocyte hyperplasia. Samples were stained by IHC to demonstrate that *F. tularensis* was present in the analysed tissue (Fig. 4, bottom/left).

**Table 2.** Pathology scoring for rats challenged by aerosol with OR96-0246

	c.f.u.	231	231	231	231	8,270	8,270	8,270	8,270	**19,200**	**19,200**	**19,200**
	**Disposition**	FD*	M/E†	Surv‡	Surv	FD	FD	Surv	Surv	FD	FD	M/E
	**TTD**	8 d	10 d	21 d	21 d	8 d	8 d	21 d	21 d	6 d	6 d	7 d
**Organ**	**Lesion**	**Score**										
Lung	Necrosis, alveolus	3	4	1	1	4	4	0	1	3	3	3
	Inflammation alveolus	3	3	2	4	2	4	5	4	4	5	5
	Inflammation perivascular	3	0	2	1	2	2	3	3	2	2	2
	Accumulated fibrin/oedema	3	4	1	0	4	4	2	3	4	4	4
	BALT hyperplasia	2	0	1	2	1	2	1	0	1	1	0
**Spleen**	**Lesion**	**Score**										
	Necrosis, lytic red pulp	2	2	0	0	3	3	0	0	2	3	3
	Necrosis, lytic white pulp	1	2	0	0	2	2	0	0	1	2	2
	Inflammation mononuclear white pulp	1	2	1	2	2	3	2	2	3	3	3
	Inflammation red pulp	3	4	2	2	3	4	3	2	4	4	4
	Accumulation of white pulp	3	2	0	0	3	3	0	0	2	2	2
	Accumulation of red pulp	4	2	0	0	3	4	0	0	3	3	3
	Lymphoid depletion	4	3	0	0	4	5	0	0	4	4	4

Histopathology lesion severity scoring scale is based upon the following scale:

0. Lesion not present.

1. Minimal (involving <10% of the tissue).

2. Mild (involving 11 to 25% of the tissue).

3. Moderate (involving 26–50% of the tissue).

4. Marked (involving 51–79% of the tissue).

5. Severe (involving ≥ 80% of the tissue).

*Found dead, less than 4 h.

†Moribund, met criteria for euthanasia intervention.

‡Survived to end of study.

Lesions in the spleen consist of lytic necrosis in the red pulp characterized by multifocal areas of karyorrhectic and other cellular debris, which were often admixed with macrophages and fewer neutrophils (Fig. 4, right). There was also necrosis in the white pulp, but it consisted predominantly of apoptosis of individual cells. Inflammation was present throughout the red and white pulp, which consisted predominantly of macrophages and neutrophils. Other findings included accumulation of haemorrhage and fibrin in the white pulp (predominantly in the marginal zone), accumulation of fibrin in the red pulp, lymphoid depletion in the white pulp and fibrin thrombi in small and intermediate-sized vessels as well as extramedullary haematopoiesis (EMH). The severity of lytic necrosis in the red pulp and inflammation in the red and white pulp were slightly greater in animals that succumbed or were euthanized in the two higher dose challenged groups. Fibrin thrombi and EMH were less consistent in the low-dose groups. The other lesions were relatively consistent across all challenge groups. Lesions present in survivors include inflammation in the red and white pulp (data not shown). Once again, IHC was used to validate *F. tularensis* presence in tissue samples that were analysed (Fig. 4, bottom right).

### Modelling of OR96-0246 pathogenesis in NHPs

Using a staircase method for NHP inhalational challenge, we investigated the disease progression following small particle aerosol exposure with the OR96-0246 strain in three CMs (Table 3). The NHPs were exposed to 4.34×10^4^ or 3.26×10^5^ c.f.u. met euthanasia criteria following challenge at days 7 and 9, respectively. As expected, samples from the lung, liver and spleen all demonstrated the presence of *F. tularensis* for both NHPs (data not shown). In contrast, NHP #15, which received 2.1×10^4^ c.f.u. survived until day 20. For NHP #15, early endpoint euthanasia criteria were met on day 20 due to complications at the telemetry surgical site rather than clinical scoring due to infection. Though this NHP did survive relative to the other NHPs receiving higher doses, clinical scoring and signs of infection were still apparent, as detailed below. At the end of the study, *F. tularensis* was recovered from the lung samples for NHP #15 but not the liver or spleen (data not shown). Based upon this initial staircase method for preliminary measurements, the LD_50_ for aerosol challenge with OR96-0246 was calculated to be ~2.1×10^4^ c.f.u.

**Table 3.** Results for NHPs aerosol challenged with OR96-0246

NHP #	Sex	Weight (kg)	Dose (c.f.u.)	TTD	Lung*	LN*	Liver*	Spleen*
15	M	4.4	2.1×10^4^	n/a	4	4	0	0
16	M	3.7	3.26×10^5^	7 days	5	4	0	2
17	M	3.1	4.34×10^4^	9 days	4	5	0	2

* Scoring of lesions observed and consisted of the percentage of tissue affected by the representative *F. tularensis* lesions as described and ranged from 0 (none), <10% minimal; 2, 10–25% mild; 3, 25–50% moderate; 4, 50–75% marked; and 5, 75–100% severe.

**Fig. 5.**
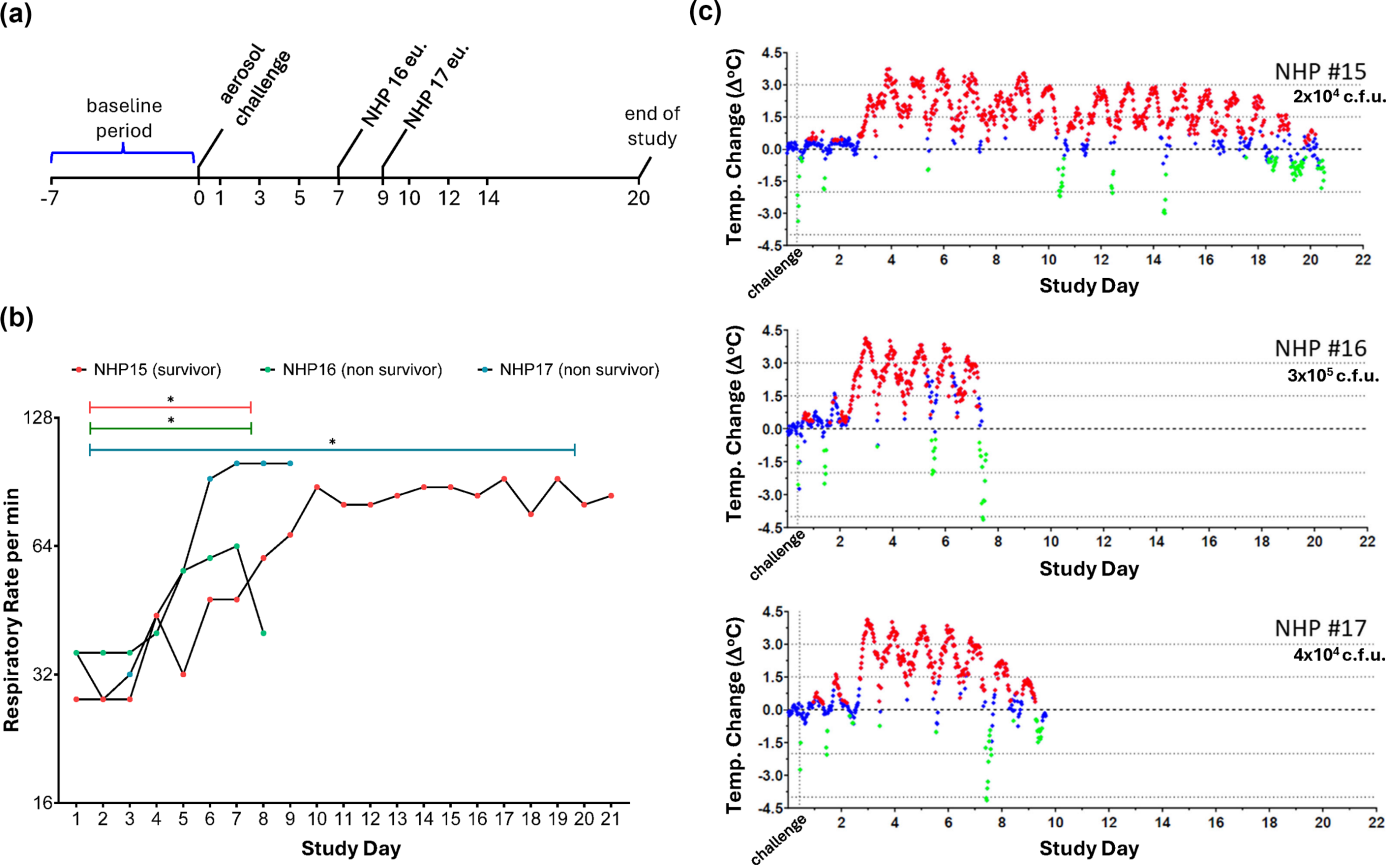
Changes in respiratory rate and body temperature in CMs exposed to aerosolized *F. tularensis* strain OR96-0246. (**a**) Schematic of study timeline in CM model. (**b**) The respiratory rate changed significantly after infection (two-way ANOVA, *P*<0.05 as *). Horizontal bars denote statistically significant respiratory rate changes after challenge compared to baseline, grouped by day 6, 7 and 20 (terminal timepoints). (**c**) Running plot of the 30 min averages of the body temperature changes for individual subjects. Body temperature changes (°C). Values −3 sd (green diamond) or +3 sd (green diamond) from baseline are considered significant; values <3 sd (green diamond) are not significant. Subjects are listed in the order of ascending target challenge dose within each group.

Following each challenge, the NHPs were monitored for clinical signs of disease as well as respiratory rate, temperature and haematology throughout the study period (Fig. 5a). The respiratory rate of all animals increased after 3–4 DPE compared to baseline, regardless of aerosol challenge dose (Fig. 5b). The average respiratory baseline (breaths per minute, bpm) for NHP #15 was 33.3; NHP #16 was 34.7 and NHP #17 was 34 bpm. While all NHPs experienced an increase in respiratory rate following challenge, the response varied between the challenge doses with respect to the respiratory range. Over the course of the infection, the respiratory rate increased 2.53 times for NHP #15, 1.84 times for NHP #16 and 2.94 times for NHP #17 when compared to the baseline (Fig. 5b). Daily clinical observations noted respiratory signs to include nasal flare and cough in all animals starting at 4 DPE. Other respiratory clinical signs were observed less frequently, with respiratory noise only noted in two animals (NHP #16 and 17) on the terminal study day. Non-descript clinical signs, such as piloerection, were noted in all animals beginning at study day 1 and continued until moribund or at the end of the study.

Telemetry was also used to provide a detailed picture of the NHP temperature over the course of the disease. Prior to exposure, all NHPs displayed normal diurnal variation in body temperature with general increases during the light cycle and decreases during the dark cycle for the period of baseline telemetry monitoring. Following exposure, disruption of this pattern was observed in all NHPs as temperature elevation, indicating a fever started between 2 and 3 days post-challenge (Fig. 5c). The exposure dose did not appear to affect the onset of sustained temperature elevation, fever or hyperpyrexia but rather changed the intensity and the peak response of the temperature changes in most subjects.

Haematology, including complete blood chemistry and cell count, was also monitored throughout the study period, after which data were analysed for trends collectively (*n*=3 to study day 7). Liver enzymes (ALT, AST, ALP; *P*<0.01) and blood urea nitrogen (BUN; *P*<0.05) significantly increased at 7 DPE, while albumin levels significantly decreased (*P*<0.05) (Fig. S1, available in the online Supplementary Material). At 5 and 7 DPE, white and red blood cell counts decreased significantly along with associated metrics (lymphocytes, monocytes, eosinophils and haemoglobin, haematocrit and platelets); however, neutrophils were significantly increased at three before decreasing below baseline levels by 7 DPE (*P*<0.05) (Fig. S2). For the surviving NHP, bloodwork returned to levels not significantly different from the baseline by Day 20.

**Fig. 6.**
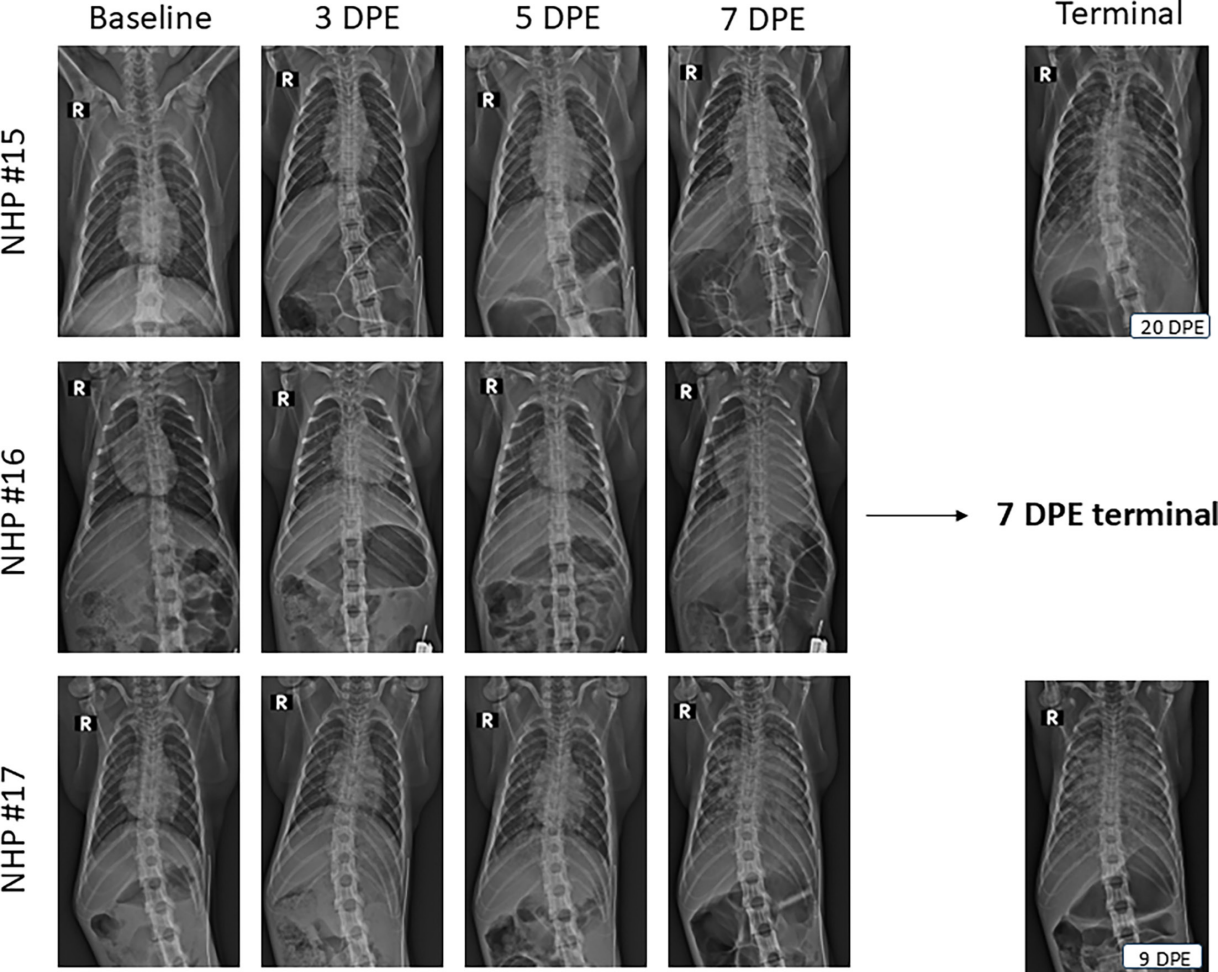
Radiographs of CMs following aerosol challenge with OR96-0246. Radiographs from 3 NHPs were taken before bacterial exposure (baseline), various DPE and terminal. NHP #15 radiographic changes begin as small, ill-defined opacities that coalesce with disease progression, terminating with a multilobular pattern. NHP #16 radiographic changes begin with a focal increased opacity that progresses to a diffuse consolidation of the left lung lobes (hemithorax). NHP #17 radiographic focal increased opacity that rapidly progresses to diffuse, ill-defined nodules and finally to large areas of consolidation at termination.

Radiographs were taken at baseline and on the indicated days post-challenge (Fig. 6). Initial radiographic changes were noted on study day three for all NHPs. Generally, radiographic changes increased in severity over time, consistent with consolidation of anterior lung lobes and increased perihilar opacity, suggesting tracheobronchial and mediastinal lymph node enlargement (Fig. 6). Non-survivor NHPs progressed to a severe alveolar pattern and consolidation of significant portions of all lung fields with air bronchograms present at study termination. NHP #17 displayed the most significant radiographic changes of all subjects. There were multiple radiographic opacities across all lung fields, coupled with the least amount of air present prior to study termination.

### Pathology of OR96-0246 aerosol-challenged NHPs

**Fig. 7.**
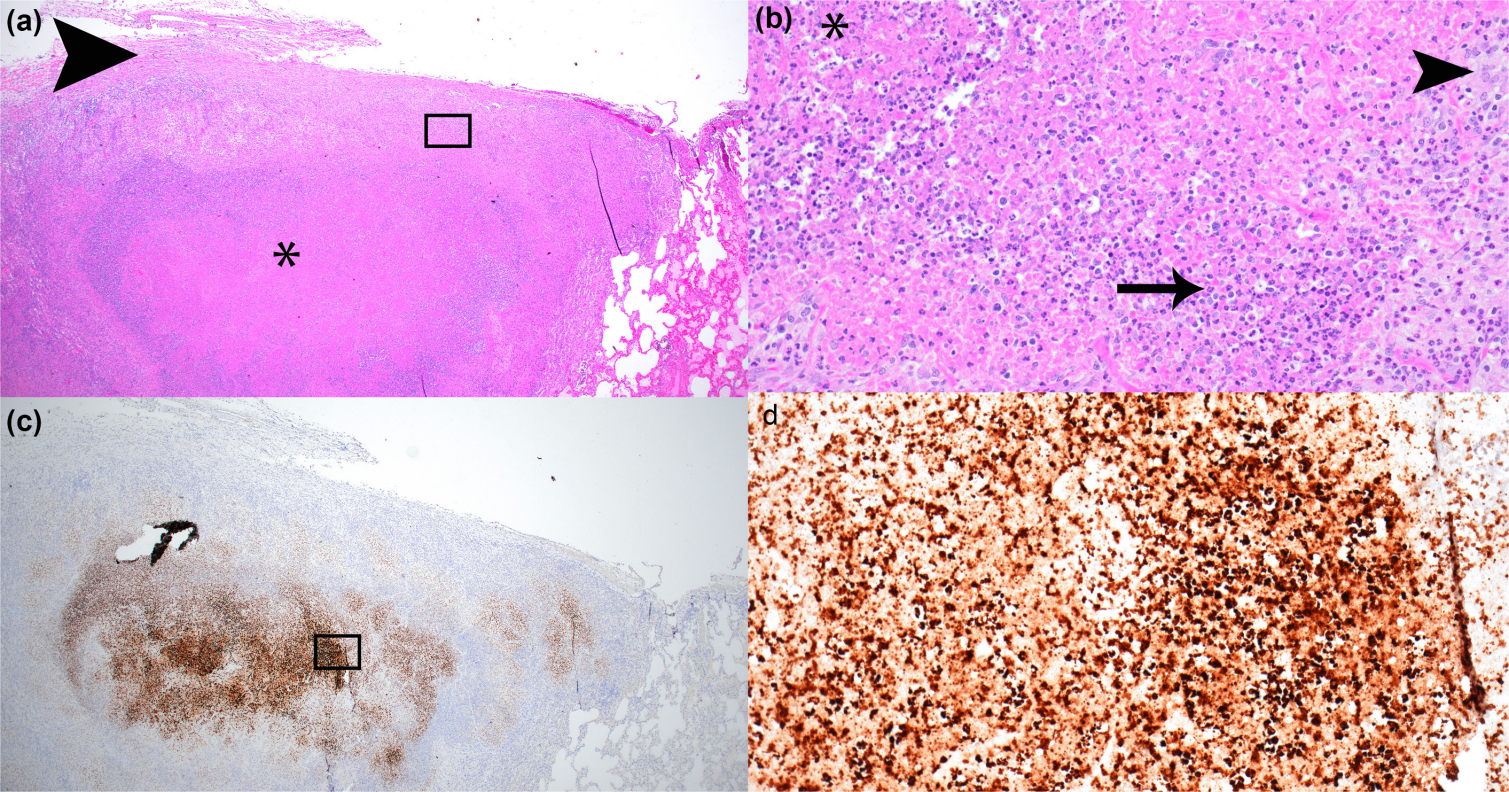
Histopathological analysis of the lungs of aerosol-challenged CMs with *F. tularensis* strain OR96-0246. NHP #17 (non-survivor) inhaled a calculated dose of 4.3×10^4^ c.f.u.(**a**) There is necrotizing bronchopneumonia (asterisk) and pleuritis (arrowhead). HE 2×. (**b**) Normal lung parenchyma is disrupted and replaced with cellular debris (asterisk), neutrophils (arrow) and macrophages (arrowhead). HE 20×. (**c**) IHC 2× and (d) IHC 20×. There is IHC positivity in the necrotic areas of the lung, indicating the presence of *F. tularensis*. The black boxes in 2× images indicate the region magnified in the 20× images.

**Fig. 8.**
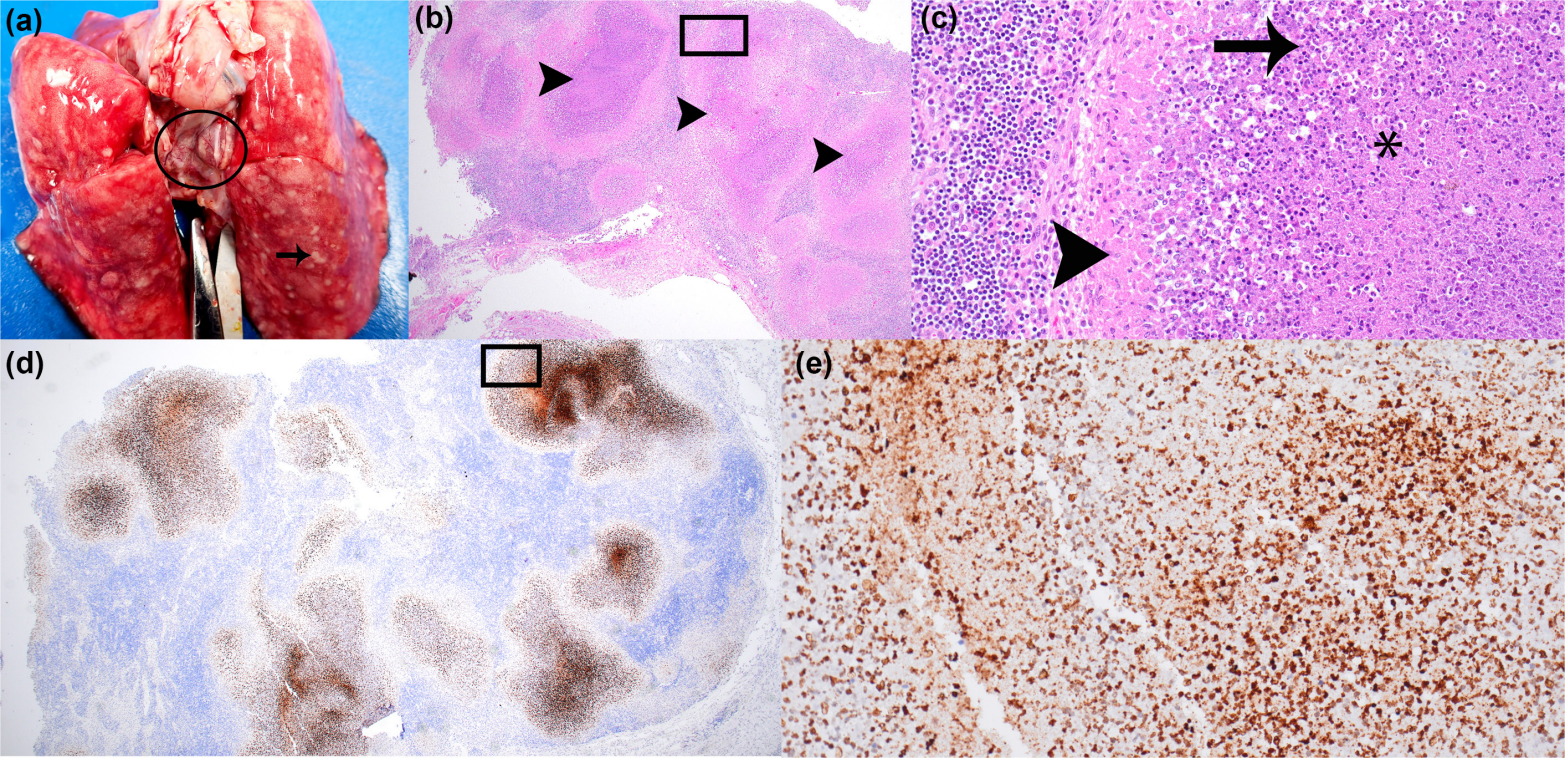
Gross pathology and histopathological analysis of the mediastinal lymph nodes of aerosol-challenged CMs with *F. tularensis* strain OR96-0246. NHP #15 (survivor) inhaled a calculated dose of 2.1×10^4^ c.f.u. (**a**) Tracheobronchial lymph node and lung. The lung lobes are severely non-collapsing, dense, red and contain many 0.3–0.5 cm white lesions (arrow). The tracheobronchial lymph node is five times its normal size (circled). (**b**) There is multifocal severe necrosis (arrowheads) of the lymph node with depletion of lymphocytes and disruption of normal architecture. HE 2×. (**c**) There is cellular and karyorrhectic debris (arrow) and a few neutrophils and macrophages (arrowhead). (d) IHC 2× and (e) IHC 20×. There is IHC positivity in the necrotic areas of the mediastinal lymph node, indicating the presence of *F. tularensis*. The black boxes in 2× images indicate the region magnified in the 20× images.

**Fig. 9.**
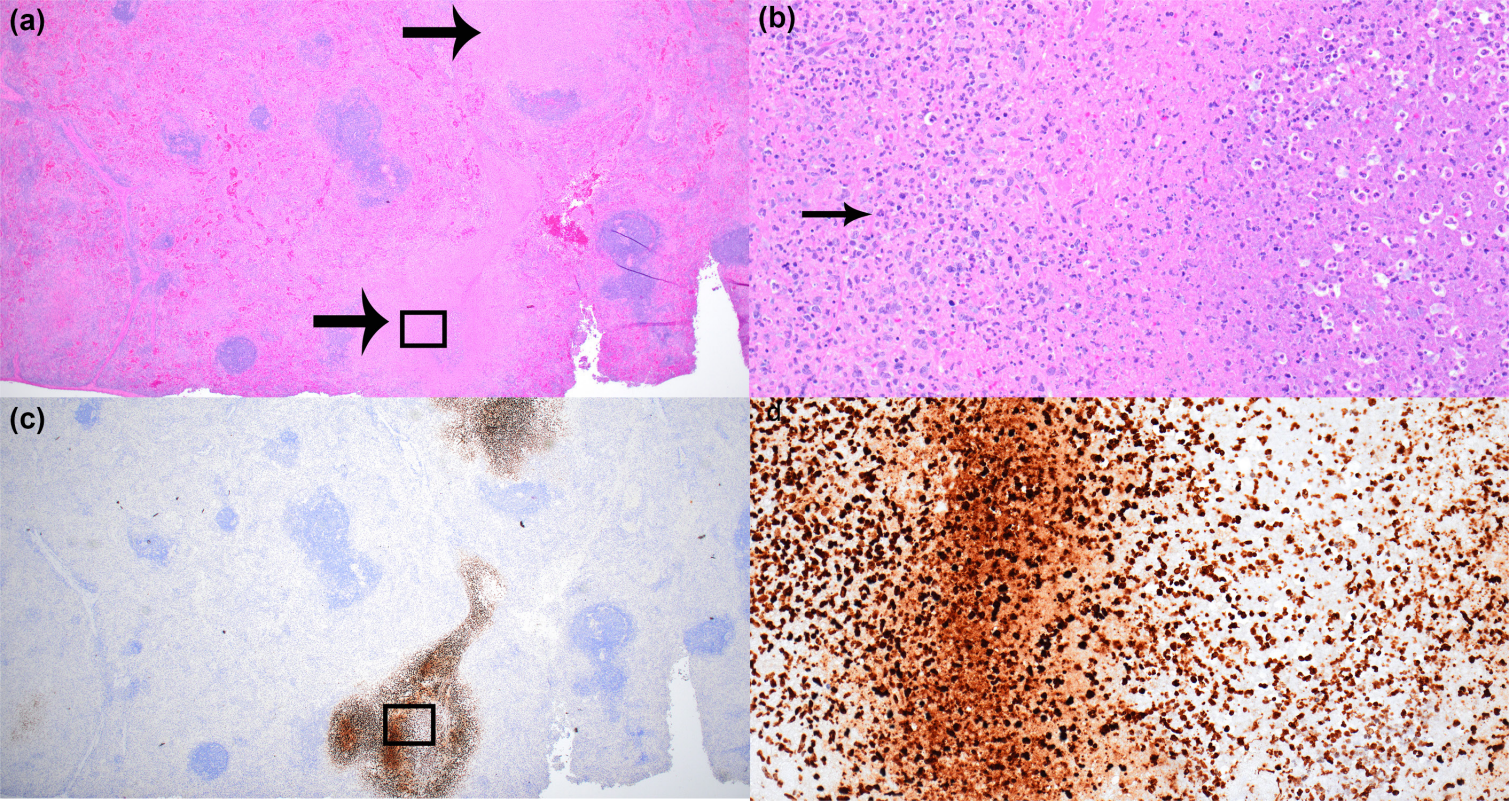
Histopathological analysis of the spleens of aerosol-challenged CMs with *F. tularensis* strain OR96-0246. NHP #16 (non-survivor) inhaled a calculated dose of 3.26×10^5^ c.f.u. (**a**) There is multifocal necrosis characterized by pallor and lack of normal architecture (arrows). HE 2×. (**b**) There is× cellular and karyorrhectic debris and a few neutrophils (arrow). HE 20×. (**c**) IHC 2× and (d) IHC 20×. There is IHC positivity in the necrotic areas of the spleen, indicating the presence of *F. tularensis*. The black boxes in 2× images indicate the region magnified in the 20× images.

 CMs are an ideal animal model for mimicking human tularemia in multiple organ systems. For the three NHPs challenged with the OR96-0246 strain, all animals showed some level of pathology, even the survivor (Table 3). Pathological changes in the NHPs were observed primarily in the lung (Fig. 7), tracheobronchial lymph node (TBLN) (Fig. 8) and spleen (Fig. 9). At necropsy, gross changes in the lungs of all three NHPs included non-collapsing and red pulmonary tissue with multifocal variably sized 0.5–1 cm well-demarcated, oval, pale, raised lesions randomly present in all lung lobes (Fig. 7a). TBLN was enlarged up to five times normal size (data not shown). The spleens of one NHP (#16) contained many 0.1–0.5 cm white, raised lesions that often coalesced (data not shown).

Histological changes in the three NHPs were noted in the lungs (Fig. 7), TBLNs (Fig. 8) and spleens (Fig. 9) of all animals. These consisted of well-delineated areas of parenchymal degeneration and necrosis with loss of architecture, cellular debris and varying amounts of haemorrhage, oedema, fibrin and vasculitis. IHC of target tissues was performed. *F. tularensis* IHC positivity was identified in the lung (Fig. 7d, e), TBLN (Fig. 8c, d), mesenteric LN and spleen (Fig. 9c, d).

Histological lesions were particularly prominent in the lung and lymphoid tissues. In the lung (Fig. 7), an association of these lesions with small and medium airways (bronchopneumonia) was apparent in less affected areas; however, as the lesions became more extensive, the association with the airway was obscured. Lung parenchyma surrounding areas of necrosis was moderately to markedly congested, and alveolar spaces were often flooded with oedema or haemorrhage. Additionally, the lung pleura over affected areas was often necrotic or fibrotic and contained variable amounts of haemorrhage, oedema and inflammation. The TBLN of all NHPs, including the survivor, contained multiple necrotizing lesions that disrupted 50% or more of the normal architecture, rupturing node capsule and spilling into the mediastinum causing perinodal mediastinitis (Fig. 8). In NHP #16, the splenic architecture, including the white pulp, red pulp and capsule, was multifocally disrupted, effaced and replaced by necrosis (Fig. 9). There was diffuse moderate to marked lymphoid depletion of the white pulp. The less severely affected splenic red pulp was markedly congested.

## Discussion

The Schu S4 strain is typically regarded as the fully virulent prototypic type A strain of *F. tularensis* that is used to gauge the efficacy of developing medical countermeasures to combat tularemia. Our laboratory has focused on developing a panel of additional *F. tularensis* strains for testing future tularemia vaccines to confirm protection beyond the prototype Schu S4 strain [36,51] and to further expand the representation of type B strains. Though type B strains are less virulent than type A strains of *F. tularensis* [51], they generally are not as well studied by the biodefence community. The most studied type B strain would be the live vaccine strain (LVS), which has served as a model surrogate of *F. tularensis* strain for research purposes, but LVS was rendered avirulent for use as a vaccine by serially passaging a strain of unknown origin [76,77]. Various research has been performed to characterize virulent type B strains of *F. tularensis*, such as epidemiological studies, antibiotic sensitivities, proteomic profiles, virulence comparisons and therapeutic treatments, to name a few examples [78,85]. But limited work has been completed in animal modelling with type B strains for testing future vaccines to protect against a wide range of *F. tularensis* strains [34,86]. Therefore, we have chosen to pursue the OR96-0246 *F. tularensis* strain as a potential prototypical type B isolate for use in future studies and aimed to construct a framework of animal modelling data for this strain.

In a previous vaccine study using rLVS Δ*capB*/*iglABC*, we noted that rats following challenge with a clinical type B strain (FRAN255) still experienced clinical signs of infection but survived a lethal aerosol challenge [36]. Certainly, this vaccine was successful in measuring survivorship after a lethal aerosol exposure. However, the goal for a successful vaccine would not only protect the military from lethal exposure but also keep them combat-ready. From our previous study, we were unsure if the issue was the particular type B strain (FRAN255) used or if the outcome was typical following challenge with any type B strain; therefore, we wished to expand our characterization and testing with additional type B strains to be better prepared for downstream testing and evaluation of potential new medical countermeasures.

The mouse model of pneumonic tularemia is a versatile model that has been used as a preliminary test mechanism of novel molecules for therapeutic intervention [87,88], treatment regimens [89], host immune response [90,91] and vaccine potential [92,93]. Further, murine models are likely the most accessible mammalian system available for many research institutes. In this particular experiment, intranasal inoculation was pursued, as this is also a lower barrier to entry for medical countermeasure development than aerosol exposure and could easily be recapitulated across laboratories. Here, we demonstrated that OR96-0246 has an LD_50_ of ~4 c.f.u. by day 7 and ultimately results in the expected 1 c.f.u. LD_50_ within 14 days by intranasal challenge. These results are consistent with other studies, as mice could not differentiate virulence between subspecies [33,94]. However, it was noted that virulent type B isolates exhibit an extended TTD relative to type A isolates, which seems to be generally consistent with the OR96-0246 strain in murine models [20,95].

The Fischer 344 rat model can potentially address nuanced virulence differences between subspecies of *F. tularensis*, as this model offers a wider dynamic range of challenge doses [96]. In this model, performed by different investigators, challenge inoculum was delivered intratracheally [46], by nose-only aerosol exposure [35,96] and whole body aerosol exposure [36] to determine the LD_50_ of various *F. tularensis* isolates. In a previous study, a direct comparison of OR96-0246 and Schu S4 by intratracheal instillation demonstrated that the LD_50_ of OR96-0246 was several logs higher than that of Schu S4 (10^5^ compared to 5×10^2^ c.f.u.), consistent with type A and B differences in virulence [46].

However, in our current study, whole-body aerosol exposure yielded an LD_50_ much lower than expected for OR96-0246, given that this strain is type B (1.3×10^2^ c.f.u.). In a previous study, we found the LD_50_ for Schu S4 by whole body aerosol exposure to be ~4.2×10^2^ c.f.u. [36] which is comparable to the Schu S4 LD_50_ values reported for intratracheal challenge (5×10^2^ c.f.u. [46]) but differs from nose-only aerosol exposure (<1 c.f.u. [35]). We speculate that these differences in LD_50_ determinations could be due to several factors, such as the growth medium used to propagate challenge material, deposition of bacteria in the lungs from the route of exposure and particle sizes generated or methods used to determine the concentration of inhaled bacteria between the various studies. In our previous Schu S4 study, we used supplemented BHI to prepare challenge material since this medium has been described as more closely mimicking a host-adapted state [67,97]. While in this current study with OR96-0246, we used commercially provided CDM to optimize standardization in growth material. Also, our inhaled doses to determine LD_50_ values are based upon sampling the impinger concentrations following exposure. In contrast, others determine inhaled doses from plating lung homogenates of specimens collected immediately post-exposure [35,98]. In the nose-only exposure study by Hutt *et al*., which described a lower LD_50_ value than was previously observed for Schu S4 in Fischer 344 rats, challenge material was also reported to be grown in CDM [35].

Previous work by others has demonstrated mixed results in how the growth material may affect the reproducibility and LD_50_ values in animal studies with *F. tularensis* [67,99, 100]. One of the most controlled studies to address these issues was using LVS to aerosol challenge mice with the bacteria prepared in various broth types (supplemented Mueller–Hinton, CDM or BHI) [99]. From this study, no differences in virulence were detected; however, BHI-grown bacteria were found to have a better spray factor following aerosolization. Taking into consideration the results of our current study with OR96-0246, additional testing may be warranted to better understand these differences for assessing LD_50_ values. Certainly, these studies further point out the need to standardize the methods for preparing *F. tularensis* challenge material, aerosolization methods and determining the delivered challenge dose for future medical countermeasure testing.

When comparing pathology from Fischer rats challenged by aerosol exposure to the OR96-0246 strain, the lesion profiles we describe here are similar to what has been reported using other *F. tularensis* challenge strains [35,36]. In general, the lesion profile for the rats challenged with OR96-0246 is similar to what Hutt *et al*. reported in the natural history study of rats aerosol challenged (nose only) with Schu S4, which described fibrinous and necrotizing lesions with lymphoid depletion [35]. Those rat lung and spleen lesions appear very similar to the samples from our study with OR96-0246 at the end of the study (euthanized when moribund). One limitation of our current study is that histopathology was limited to the lung and spleen. Based on the prior studies with F344 Fischer rats exposed to Schu S4 as reported by Hutt *et al*. [35], lesions are not limited to only the lungs and spleen. Affected tissues may also include the nasal cavity, mandibular, tracheobronchial and mediastinal lymph nodes, as well as the liver, bone marrow and gastrointestinal tract, among other organs. Specifically, lymph node lesions often present as fibrinous and/or necrotizing inflammation. Liver lesions typically demonstrate neutrophilic and/or histiocytic inflammation and may include necrosis depending on the sampling timeframe. Histiocytic inflammation with fibrinous/necrotizing components has been observed in bone marrow, and necrotic foci may appear in the small intestine. Inflammation has also been reported in other organs, such as the heart and kidneys. In summary, the rat histopathological findings with OR96-0246 are very similar to what was reported previously with other *F. tularensis* strains.

The O-antigen plays a vital role in host immune subversion and has been linked to the ability to stochastically form biofilm [58,101, 102]. In our assays, OR96-0246 displayed canonical O-antigen by western analysis but more readily formed robust biofilm compared to Schu S4. These data are consistent with our previous observation that type B isolates appear to vary more rapidly than type A isolates [58]. While this is likely indicative of a strain’s propensity to vary the O-antigen, the role of this variation, if any, remains unknown. O-antigen variants maintain intracellular invasion efficacy but display poor intracellular replication [75,103], perhaps entering a temporary carrier state within host cells and exhibiting decreased virulence [104]. Additionally, it remains unclear if biofilm formation in *F. tularensis* can influence host range or enable environmental persistence [74], while traditionally, biofilm formation enables bacteria to cope with environmental stressors. Not surprisingly, the concentrations of select components within the medium affect the growth rate and cell density [105,107]. Further, type A strains have been shown to reach a higher density than type B isolates, except LVS [108]. Consistent with our previous studies, a more robust cell growth was observed in CDM as compared to cultivation in supplemented BHI [51]. However, media selection can also help mimic host adaptation *in vitro* and perhaps prime bacterial cells for a pathogenic state [67,97, 109]. We found that increasing IsoVitaleX had little effect on OR96-0246 growth in BHI, while significant increases were observed for Schu S4 (Fig. 1). For these reasons, we continued to propagate OR96-0246 in supplemented BHI for NHP modelling to maintain consistency with other datasets generated with *F. tularensis* strains from the challenge panel strain (unpublished).

 The CM has emerged as the primary NHP model for pneumonic tularemia using Schu S4 as aerosol challenge material [39,41, 47, 48, 110]. In these studies, the NHP infectious dose and pathophysiology have more closely resembled clinical disease progression in humans infected by a type A isolate [40,41]. We expand upon the testing capabilities available to the research community by utilizing OR96-0246 to describe type B virulence and disease progression in the CM tularemia model. Consistent with expectations for type B isolates, we found that OR96-0246 had a higher LD_50_ (~2×10^4^ c.f.u.) than the Schu S4 strain (20 c.f.u. [44]), which suggests that CMs do indeed reflect general human susceptibility to tularemia.

However, we do note that this LD_50_ value is based upon a staircase LD_50_ method, and the NHP, which survived until day 20, still had clinical signs consistent with pulmonary disease. Euthanasia intervention occurred on day 20 due to complications experienced at the telemetry surgical site and not based upon disease clinical scoring. We cannot rule out that if the study was carried out for a longer time frame (>21 days), the NHP could have reached clinical scoring for euthanasia intervention due to the disease, which would affect the LD_50_ calculation. However, an increase in survival time (20 DPE) was certainly reached as compared to those NHPs that received higher c.f.u. doses (7- and 9 DPE). Given the limited number of NHPs currently used here, a more complete study with additional animals centred around this initial estimate would provide a better LD_50_ value for this animal model. But the current NHP study does serve as the framework of a model for future development and medical countermeasure testing of tularemia with a type B strain.

The telemetry data of the NHPs challenged with OR96-0246 mirrors the disease course that was previously observed for Schu S4-challenged NHPs, as the febrile period began ~2.5 days post-infection and persisted throughout the study period [39,41]. Furthermore, this is generally in line with the acute onset of fever in clinical reports of tularemia [1,111]. Additionally, OR96-0246 induced an increase in the respiratory rate of the NHPs during the febrile period, which was also observed in Schu S4-challenged CMs, suggesting that the outward disease presentation is consistent between type A and B isolates. Haematology and blood chemistry are generally consistent with aerosolized exposure in other NHP models, as CMs exposed to Schu S4 developed thrombocytopenia, lymphopenia and increased liver enzymes following the challenge [39]. Future studies will be required to understand if OR96-0246-challenged animals respond similarly to medical countermeasures as those exposed to Schu S4.

A previous pathological aerosolized tularemia challenge study examined African Green (*Chlorocebus aethiops*) monkeys (AGM) aerosol challenged with Schu S4 [112]. Generally speaking, the types of necrotic lesions observed were similar between this previous study and our current study with CMs aerosol challenged with OR96-0246. However, some important differences were noted between these two studies. The splenic lesions of the CMs infected with OR96-0246 were only mild (Fig. 9) in contrast to splenic lesions, which were more pronounced and frequently moderate to marked, observed for the AGMs challenged with Schu S4 [112]. It is not possible to discern the reasons for these differences since different species of NHPs were used in addition to different *Francisella* subspecies. However, it is well established that type A isolates are more virulent than type B isolates, and this likely accounts for the difference in severity of lesions found in the spleen. Furthermore, in a previous study that performed a side-by-side progression comparison of tularemia following aerosol challenge with Schu S4 between three NHP species, it was demonstrated that the CMs most consistently manifested pathological responses from infection similar to human infections [40].

 Although two of the three CMs succumbed to disease within 7–9 days, one animal challenged with 2×10^4^ c.f.u. of OR96-0246 survived but was euthanized at the end of the study (day 20). However, this animal also had significant pathology (Fig. 7). This ability of the animal to survive until day 20 may also be due to diminished virulence for type B isolates as compared to type A strains of *F. tularensis*. The time to death for Schu S4 by aerosol challenge is 6–10 DPE in AGMs, CMs and rhesus monkeys [39,40, 112]. The previous study, which focused on the natural history of CMs challenged with Schu S4, did not include a full set of tissues for a complete evaluation and comparison to our current study with OR96-0246. Future studies with CMs challenged with OR96-0246 would benefit from the evaluation of all tissues, as lesions can be observed in the gastrointestinal tract, genitourinary, endocrine, cardiovascular, ophthalmic and nervous systems in human and NHP species [40].

 To allow for additional medical countermeasure testing and verification of results, we wished to use a type B strain that would be available to other biodefence research laboratories. In addition, it would be desirable to have the genome sequence of the test strain. From this perspective, the *F. tularensis* subsp. *holartica* strain OR96-0246 would appear to be ideal as the strain is available from BEI Resources to qualified BSL-3 laboratories, and the sequence of the genome has been completed [113]. Also of benefit, a transcriptomic study has been completed with the OR96-0246 strain, and the results were compared to the prototype Schu S4 strain [114]. From this study, interesting results were identified from comparing the differing levels of transcript expression from these two strains, which may explain the virulence differences between *F. tularensis* type A and B subspecies.

Notably, high levels of expression were noted in an operon of Schu S4, which encodes genes for the synthesis and transport of isochorismatases. In contrast, one of the ORFs in the operon in OR96-0246 was interrupted with a transposase [114]. Typically, these gene products are involved in sequestering iron, but their exact role in *Francisella* remains to be determined [115]. Additionally, other Schu S4 genes (*FTT_0127*, *FTT_0442*, *FTT_0707* and *FTT_1004*), which encode for predicted transporters, had higher expression levels compared to OR96-0246. The gene *FTT_1234* encoding a putative choloylglycine hydrolase (CGH) was found to be more highly expressed in Schu S4. These enzymes have been considered to have some role in virulence in other pathogenic bacteria by helping the bacterium circumvent the antimicrobial properties of bile salts [116,117]. For example, in *Listeria monocytogenes,* the deletion of the CGH gene *bsh* is associated with a decrease in colonization of liver tissue, while in *Brucella abortus,* the deletion of *cgh* impaired intracellular replication *in vitro* [116,117]. It is noted that the orthologue in OR96-0246 contains a transposon insertion upstream, which may lower its expression. Differences in gene expression (in addition to previously demonstrated lack of genes) involved in polyamine synthesis were also detected. Additional genes upregulated in type A vs. type B isolates also included *mdaB*, a gene encoding for a NADPH-quinone reductase; *tlyC*, a gene which is annotated as a haemolysin, but its exact function is unknown since no haemolytic activity has been detected; *pdpD*, a gene present in the Francisella pathogenicity island; and *pilT* and *pilA*, genes which encode for pilus proteins. In contrast, other genes were transcribed at higher levels in OR96-0246 compared to Schu S4; these included the orthologs of several hypothetical proteins (FTT_0185, FTT_0845 and FTT_1143). In addition, the *FTT_0642* gene, which encodes a gene involved in valine biosynthesis, was also expressed at a higher level in OR96-0246.

 We have characterized OR96-0246, a type B strain of *F. tularensis*, for future testing of medical countermeasure development to confirm protection against type B strains rather than just exclusively the prototype Schu S4 type A strain. Successful tularemia medical countermeasures would need to protect against a variety of *F. tularensis* strains of differing origins and virulence attributes. The animal modelling described here with this type B strain provides a framework for future testing with established tularemia models to better assess future vaccines and therapeutics during the transition and maturation process for product development to obtain regulatory approval.

## Supplementary material

10.1099/mic.0.001637 Uncited Fig. S1.

## Abbreviations

AGI: all-glass impinger
AGM: African green macaque
BEI: Biodefense and Emerging Infections Resources
BHI: brain heart infusion
bpm: breaths per minute
CDM: Chamberlain’s defined media
CGH: choloylglycine hydrolase
CM: cynomolgus macaque
DPE: days post-exposure
DSI: Data Sciences International, Inc.
EMH: extramedullary haematopoiesis
IHC: immunohistochemistry
LVS: live vaccine strain
NHP: non-human primate
TBLN: tracheobronchial lymph node
TTD: time to death
