# Supplementary material for: Animal modelling with the Francisella tularensis subspecies holarctica strain OR96-0246
Source: Microbiology (Reading). 2025 Dec 18;171(12):001637. doi: 10.1099/mic.0.001637 (PMC12713684; doi:10.1099/mic.0.001637)
Supplement: Uncited Fig. S1. [file mic-171-01637-s001.pdf]

Animal modeling with the *Francisella tularensis* subspecies *holarctica* strain OR96-0246

Supplementary Material

Kevin D. Mlynek[1*]; Sara I. Ruiz[1]; Curtis R. Cline[2]; Alexandra N. Jay[3]; Ju Qiu[4#]; Ronald G. Toothman[1]; Elsie E. Martinez[1]; Wannaporn I. Ittiprasert[1^]; Nancy A. Twenhafel[2]; Joel A. Bozue[1*]

[1]Bacteriology Division, [2]Pathology Division, [3]Veterinary Medicine Division, [4]Regulated Research Administration Division, U.S. Army Medical Research Institute of Infectious Diseases (USAMRIID), Frederick, MD, USA

*Corresponding authors:
kevin.d.mlynek.civ@health.mil
joel.a.bozue.civ@health.mil

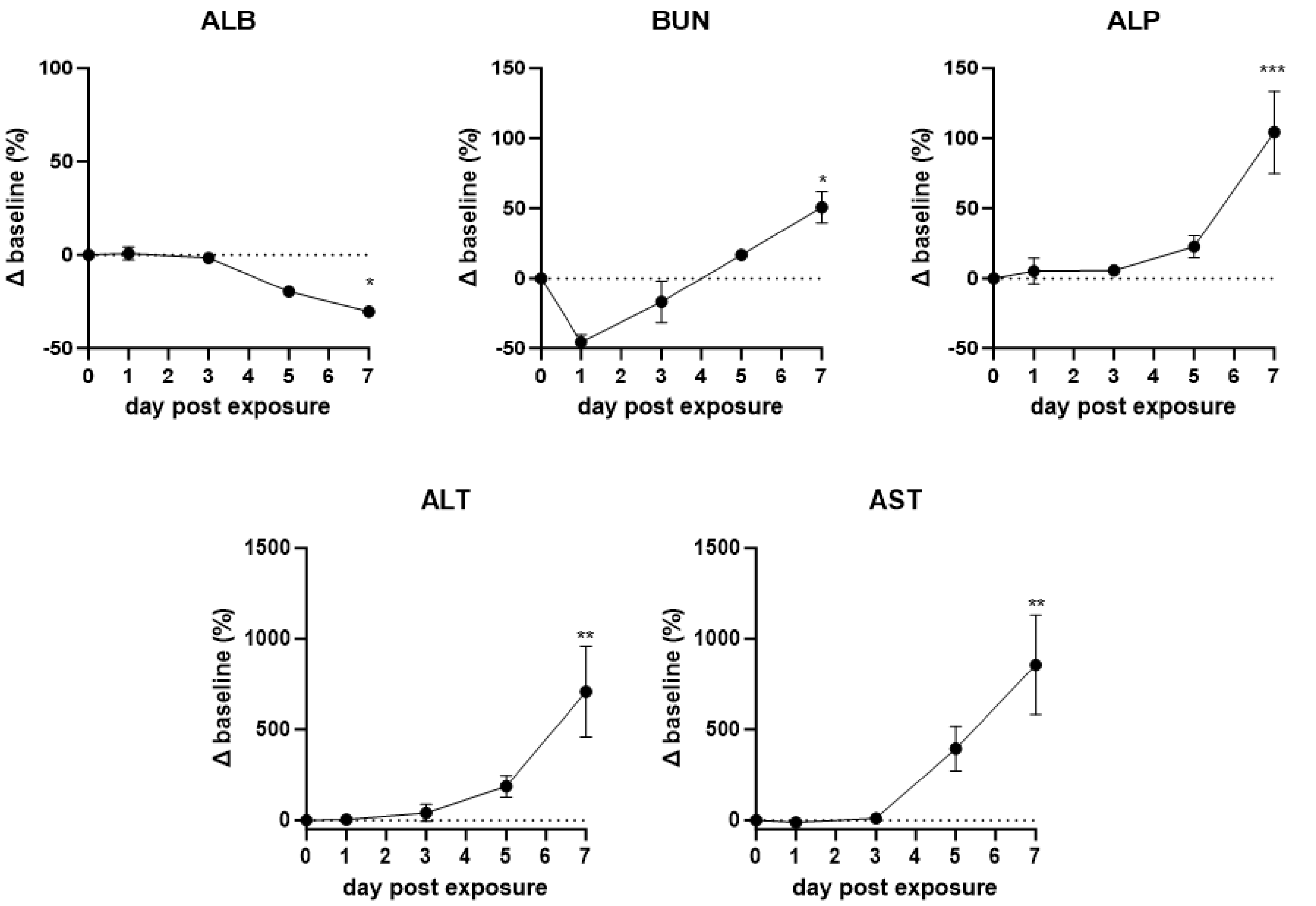

Figure S1. Significant changes in NHP blood chemistry following infection with OR96-0246. Blood was obtained at a baseline reading as well as 1, 3, 5, and 7 days post exposure for analysis. Significant changes in circulating levels relative to the baseline sampling were identified for albumin (ALB), blood urea nitrogen (BUN), alkaline phosphatase (ALP), alanine aminotransferase (ALT), and aspartate aminotransferase (AST). *$P<0.05$, **$P<0.01$, ***$P<0.001$

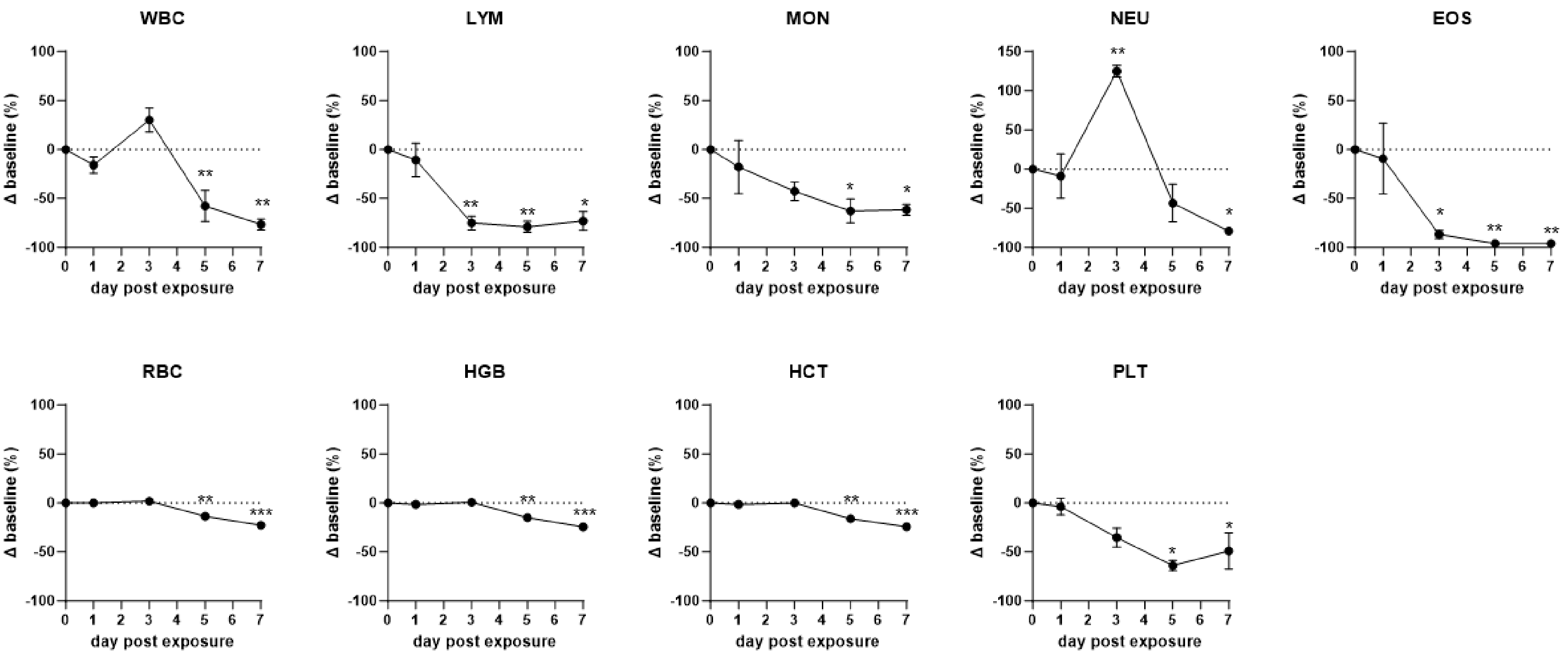

Figure S2. Significant changes in NHP complete blood count panel following infection with OR96-0246. A complete blood count (CBC) was performed on samples obtained from NHPs post exposure. Significant changes in circulating levels relative to the baseline sampling were identified for white blood cells (WBC), lymph (LYM), monocytes (MON), neutrophils (NEU), eosinophils (EOS), red blood cells (RBC), hemoglobin (HGB), hematocrit (HCT) and platelets (PLT). *$P<0.05$, **$P<0.01$, ***$P<0.001$
